# A 3 pJ/bit free space optical interlink platform for self-powered tetherless sensing and opto-spintronic RF-to-optical transduction

**DOI:** 10.1038/s41598-021-87885-6

**Published:** 2021-04-19

**Authors:** Skyler Wheaton, Victor Lopez-Dominguez, Hamid Almasi, Jialin Cai, Zhongming Zeng, Pedram Khalili Amiri, Hooman Mohseni

**Affiliations:** 1 Department of Electrical and Computer Engineering, Northwestern University, Evanston, IL USA; 2 Suzhou Institute of Nano-Tech and Nano-Bionics, CAS, Suzhou, Jiangsu 215123 People’s Republic of China

**Keywords:** Optics and photonics, Micro-optics, Electrical and electronic engineering

## Abstract

Tetherless sensors have long been positioned to enable next generation applications in biomedical, environmental, and industrial sectors. The main challenge in enabling these advancements is the realization of a device that is compact, robust over time, and highly efficient. This paper presents a tetherless optical tag which utilizes optical energy harvesting to realize scalable self-powered devices. Unlike previous demonstrations of optically coupled sensor nodes, the device presented here amplifies signals and encodes data on the same optical beam that provides its power. This optical interrogation modality results in a highly efficient data link. These optical tags support data rates up to 10 Mb/s with an energy consumption of ~ 3 pJ/bit. As a proof-of-concept application, the optical tag is combined with a spintronic microwave detector based on a magnetic tunnel junction (MTJ). We used this hybrid opto-spintronic system to perform self-powered transduction of RF waves at 1 GHz to optical frequencies at ~ 200 THz, while carrying an audio signal across (see Supplementary Data for audio files).

## Introduction

Wireless interlinks are attractive for a variety of applications across medical and scientific fields. Realizing devices that can be interfaced with in a tetherless fashion opens many possibilities for implantable medical devices^1^ and environmental sensors^2,3^ that would otherwise be impossible or highly impractical. Considerable challenges that come with any tetherless system are power delivery and data collection/transfer efficiency. In any tetherless system available energy is precious and needs to be used as efficiently as possible, thus devices with the lowest energy consumption per bit of information transferred are ideal. Once physical electrical interfaces are removed, the only two options to power the device are either to include some form of local power source, or to provide power wirelessly. Devices that rely on an on-board power storage scheme still require some form of external power delivery to periodically charge them for long-term operation. Devices that require both power storage and delivery are significantly limited in their scalability and as such are not suitable for a large array of applications. A solution that has gained popularity in recent years is the use of power generators, which can locally harvest energy from the environment, such as vibrational^4^ or chemical energy^5,6^. These generators are ideal for some specific applications^7,8^ but are unideal in situations where a semi-constant energy source cannot be ensured. A more robust approach that has seen significant use in biomedical and industrial applications is the deployment of radio frequency identification (RFID) circuits that harvest provided RF energy using an antenna and a rectification circuit to power local CMOS circuitry/sensors. Passive RF-coupled tetherless devices have been demonstrated with both near-field and far-field coupling. Far-field coupled devices have been demonstrated up to a range of 6 m, with energy per bit values in the nano-Joule range^9–11^. Near-field coupled RF-devices have been demonstrated at ranges up to several tens of millimeters, with energy per bit values falling to the pico-Joule and hundreds of femto-Joule range^12–16^. The primary drawback of a wireless RF powered system is the device size restriction due to the large area being taken up by essential CMOS circuitry, and the need for relatively large antennas for effective coupling. RF energy harvesters based on spintronic devices^17^ (magnetic tunnel junctions, MTJs) have been proposed as an alternative to traditional RF power harvesters, due to their very small size (< 0.01 µm^2^) and large sensitivity to the input RF power (> 100 mV/µW)^18–20^. These novel solutions to RF rectification allow for smaller RF-powered devices; however, as the size of an RF-coupled device is reduced, the interlink becomes increasingly lossy due to the long wavelength of RF signals. When including the antenna area, the smallest near-field bi-directional devices demonstrated to date are on the order of 1 square millimeter^12^.

A solution to the drawbacks of typical RF interlinks is to apply the same methodology to a system that uses a significantly shorter wavelength. Free space optical interlinks have been demonstrated in the past in a variety of different applications and range scales. Long range optical links based on retro-reflecting surface normal modulators^21^ have been demonstrated at ranges up to 2 km. At sub-meter range scales, optically powered CMOS circuits leveraging integrated LEDs that act both as a photovoltaic and an optical emitter have been investigated for optical link applications^22–24^. The advantage of using an optical signal lies in the ability to effectively collimate and deliver optical power to a small area, this minimizes wasted power and has the additional benefit of allowing for high coupling efficiency to sub-mm scale devices. The high directionality of an optical interlink poses new beam aiming and alignment challenges over a typical RF communication link. Interlinks with retro-reflectors can address the angular sensitivity of the link, but it is still essential that the input beam be accurately aimed at the optically active region of the device. Solutions to these challenges have already been demonstrated by using real-time aiming systems to maintain proper alignment. For example, in Ref.^21^ the transmission of video from a moving aircraft to an immobile interrogator was demonstrated using an active aiming system and a retro-reflecting modulator at a range of 2–4 km. At shorter ranges, mm-scale MEMS mirror systems and galvo mirror systems have been demonstrated for active beam aiming applications^25^.

**Figure 1:**
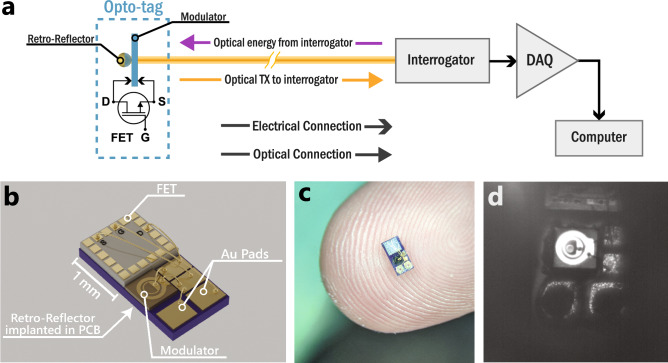
(**a**) Schematic of free space optical link test system showing the opto-tag comprised of a FET, SQWM, and a retro-reflector and the optical/electrical connections between the tag and the interrogation system. (**b**) 3D render showcasing the design of the SWIR opto-tag. (**c**) Stereo-Microscope image of the final device used in this work. (**d**) SWIR image of the mounted modulator showing the retro-reflector embedded beneath.

The emergence of CMOS devices that can be operated by harvesting optical power has opened many new possibilities for tetherless technologies; however, all demonstrations of bi-directional optically interrogated CMOS devices to date have relied on LEDs for optical absorption and emission. Optical power can be delivered to these devices with a collimated beam, but typical un-lensed micro-LEDs emit light with angular distributions on the order of 55 degrees FWHM^26^. The high angular spread of the emitted light greatly limits the max read range of these devices. In this work, we extend these concepts to produce self-powered electro-optical tags (opto-tags) that harvest energy from an input optical beam while simultaneously encoding data on it and returning it to an interrogator. A schematic of the opto-tag measurement system is shown in Fig. 1a. Low voltage operation is achieved by using stepped quantum well modulators (SQWMs) which are multi-quantum-well (MQW) devices with tiered quantum well potentials^27^. SQWMs require more complicated material growths than MQW devices, but they can be optimized to produce high modulation strength for small applied voltages^27,28^. The SQWM is hybridized with a field effect transistor (FET) which acts as a high impedance input stage of the opto-tag. The high impedance input isolates the sensitive SQWM from a potential sensor connected to the opto-tag. The transconductance of the FET produces an input gain which further increases the optical modulation that can be achieved with the SQWM for small applied gate voltages. The SQWM is also combined with a spherical retro-reflector which has been shown to work at angles of incidence up to 80 degrees and has been shown to increase the return intensity from devices implanted in scattering media^29^. The integration of a FET, SQWM, and spherical retro-reflector enables an opto-tag that has low angular sensitivity, low input referred noise, and on demand self-powered operation. In this work, a 1.2 mm by 2.2 mm self-powered opto-tag is constructed using a short wavelength IR (SWIR) SQWM. A detailed 3D schematic and a microscope image of a completed device are shown in Fig. 1b,c, respectively. A 280 µm diameter spherical retro-reflector was implanted in the carrier PCB underneath the SQWM, as shown in Fig. 1d. The SQWM used in this work was fabricated with a 300 µm diameter. Details of the SQWM structure and fabrication are provided in the supplemental information.

In the following section the opto-tag operation is analyzed, and the performance is compared to other technologies via a unifying figure of merit which rewards communication range, efficiency, and device size. The opto-tag is then combined with a nanoscale spintronic MTJ microwave detector. The MTJ consists of two ultrathin ferromagnetic layers separated by a dielectric tunnel barrier, where the input RF voltage induces a precession of the magnetization in one of the magnetic layers (i.e. the free layer), in response to spin-transfer torque (STT) and voltage-controlled magnetic anisotropy (VCMA) effects^30,31^. This creates a resistance oscillation of the device due to the tunneling magnetoresistance (TMR) effect, which in turn gives rise to a rectified DC voltage. By connecting this MTJ-based spin-torque microwave detector (STMD) to the optical tag, we demonstrate a fully self-powered RF-to-optical transducer capable of collecting signals in the RF domain and transcoding them into the optical domain for secure and tetherless collection at range. We demonstrate direct detection of amplitude-modulated (AM) signals on a 1 GHz carrier frequency using our hybrid self-powered opto-spintronic device.

## Results and discussion

The key component of the self-powered optical tag is the SQWM, which is an electro-absorptive modulator. The SQWM has a variable absorption which is modulated by an applied electric field across the device. The quantum wells are sandwiched between p and n doped capping layers that produce a PIN-like structure for efficient biasing of the device. The device used in this work was designed by a genetic optimization algorithm^27^ to work ideally with small voltage swings, on the order of several millivolts. In depth details of the SQWM design and structure are discussed in the Supplementary Fig. S1a,b. The absorption of photons within the active region of the SQWM produces a current flow which can then be used to power local electronics surrounding the modulator. The current flow produced by absorbed photons from an input optical beam is harnessed by connecting the terminals of the SQWM across the drain and source of the FET. The photocurrent generates a voltage across the resistive drain-source channel and thus across the SQWM itself, forward biasing the device. When a voltage is applied to the gate of the FET, the channel resistance between the drain and source is altered producing a change in the built-up bias voltage across the SQWM. This shift in the bias will alter the electric field across the SQWM and produce a change in the transmission. This shift in transmission is detected remotely with a photodetector which can then be amplified if needed, and finally digitized. A diagram of the opto-tag circuit is shown in the Supplementary Fig. S2a.

The gain produced by the interplay of the FET and SQWM can be described by Eq. (1),

1 $$G_{in} = \left( {\frac{{g_{m} }}{{g_{o} + g_{mod} }}} \right),$$

2 $$\Delta V_{mod} = \Delta V_{gate} G_{in} ,$$

where $g_{m}$ is the transconductance of the FET, $g_{o}$ is the output conductance of the FET and $g_{mod}$ is the conductance of the modulator. Since the characteristics of the FET are fixed, it is clear from Eq. (1) that maximum gain is achieved for any chosen FET if the conductance of the modulator is minimized, i.e., a modulator with low leakage and a large turn-on voltage is ideal. This equation assumes that both devices are operated in their respective linear regions. In practice, this requires the FET to be in saturation and the SQWM to be below the forward-bias turn on voltage. The bias point is controlled by the optical power incident on the SQWM. The FET (ALD212900) output characteristics and the illuminated SQWM IV characteristics displaying an input gain of ~ 20 are shown in the Supplementary Fig. S2b. Because the input gain is dependent on the IV characteristics of the SQWM, the performance of the opto-tag will depend on the optical power incident on the modulator. The device demonstrated here shows peak input gain when illuminated with 40 µW of optical power. The gain drops to half its peak value within a range of 10 µW around the optimum illumination power. The sensitivity to biasing is dictated by the transconductance of the FET and the illuminated IV characteristics of the SQWM, regions where both devices have the highest differential resistance will yield the highest gain. In our system the interrogator is simultaneously acting as the opto-tag remote power source and the opto-tag reader allowing a feedback loop between the sent optical power and the return signal quality to be implemented to maintain optimum link performance.

### Self-powered efficiency

Ultimately, the power consumed by the opto-tag device is the optical power that is sent to the device to operate it. The optical power absorbed by the SQWM can be tuned during growth either by altering the quantum well material compositions or by changing the number of quantum well periods in the active region of the device. The input optical power absorbed by the 47 period SQWM used in this work is ~ 20% for a single pass through the quantum well region. The absorbed optical power is consumed at the opto-tag while the transmitted optical power is consumed at the interrogator. The opto-tag is also an analog device with a full-scale output dictated by the linear operation regions of the FET and SQWM used in the design. A full-scale effective number of bits can be defined as^32^:

3 $$N_{bit} \cong Log_{2} \left( {1 + SNR_{FS} } \right)$$

where $SNR_{FS}$ is the maximum signal-to-noise ratio achievable at a given sample rate. The effective energy consumption of the interlink per bit sent is:

4 $$E_{bit} = \frac{{P_{sent} }}{{N_{bit} SR}},$$

where $P_{sent}$ is the total optical power sent to the device and $SR$ is the sample rate. The remaining question is the bandwidth limit of a potential opto-tag device. FETs have already been shown to operate easily beyond several GHz. Therefore, the inherent limitations on modulation bandwidth will come from the speed limitations of the SQWM in parallel with the resistance of the drain-source channel. The 3 dB bandwidth of the device used in this work, which is comprised of an ALD212900 FET and a 300 µm SQWM ($C_{mod}$ =  ~ 10 pF) is approximately 2 MHz. This bandwidth can be tuned by altering the thickness and diameter of the SQWM. Adding more periods to the active region of the SQWM will decrease the capacitance while increasing optical absorption. Similarly, FETs with lower drain-source resistances and smaller gate capacitances would produce higher bandwidth devices but would also require a higher optical bias power to reach optimum gain.

The maximum possible bandwidth obtainable by the opto-tag is limited by the available power harvested from the input optical beam. An upper bound on the power limited bandwidth can be calculated by assuming all of the absorbed optical power is consumed to drive the capacitance of the SQWM and the FET gate. Under these assumptions the power limited bandwidth is:

5 $$BW_{p} = \frac{{2I_{p} }}{{\left( {C_{gate} + C_{mod} } \right)\Delta V_{mod} }},$$

where $I_{p}$ is the photo generated current and $C_{gate}$ is the FET gate capacitance. $C_{gate}$ is nominally 30 pF for the ALD212900. The opto-tag presented here flows ~ 15 µW of photo current when operating at peak gain. With the chosen FET and SQWM, the highest $\Delta V_{mod}$ obtainable is ~ 150 mV. Using these values, the power limited bandwidth of the opto-tag is ~ 5 MHz.

Another important performance parameter for a tetherless device is the range at which the device can be efficiently interfaced with. For the retro-reflecting opto-tag, the range is ultimately limited by the divergence of the retro-reflected collimated beam. Assuming a diffraction-limited beam, the diameter of the beam as it propagates from the opto-tag to the interrogator optics can be described as^33^:

6 $$D_{beam} \left( z \right) = 2R_{retro} \sqrt {1 + \left( {\frac{z\theta }{{R_{retro} }}} \right)^{2} } ,$$

where z is the distance the beam has traveled after being retro-reflected from the opto-tag, $\theta$ is the divergence angle of the collimated beam, and $R_{retro}$ is the radius of the retro-reflector used in the opto-tag system. A spherical retro-modulator with a larger diameter can support a larger beam waist and thus a lower divergence. Assuming the coupling beam diameter is matched to the diameter of the retro-reflector, then the divergence angle is directly related to the retro-reflector radius by^33^:

7 $$\theta = \frac{\lambda }{{\pi R_{retro} }} .$$

Therefore, a larger retro-reflector is ideal for longer range operation. Similarly, under the same assumptions as Eq. (7), the number of nearfield modes supported by the retro-modulator can be estimated by taking the ratio of the retro-modulator active area to the spot area. At high numerical aperture the spot radius approaches $\frac{\lambda }{\pi }$ the number of modes can then be shown to be proportional to:

8 $$N_{modes} \cong \frac{{\pi^{2} R_{retro}^{2} }}{{\lambda^{2} }} .$$

From Eqs. (7) and (8), it can be shown that the number of supported nearfield modes and the divergence angle of the coupling beam are related by:

9 $$N_{modes} \cong \frac{1}{{\theta^{2} }} .$$

Because of this relationship between the divergence angle and the number of supported modes, it is informative to consider the efficiency of the optically coupled devices in terms of joules/bit/mode. For example, a single-mode waveguide modulator could potentially produce significantly higher depth of modulation than the multi-mode device presented here; however, the interrogation range would be prohibitively short because the system can only support a single mode and thus would have a very high divergence. Of course, it is possible to collimate the output of a low mode count device, but this will not alleviate the high sensitivity to small misalignments and even the slightest vibrations. A highly multi-mode device is very forgiving with alignment because the incoming optical energy can couple freely to many different modes. device presented here with a 300 µm diameter retro-reflector and a wavelength of 1550 nm, the number of supported modes is ~ 37,000.

### Self-powered hybrid opto-spintronic RF-to-optical transducer

The self-powered nature of the optical tag makes it ideal for implantable devices, or for working with other sensors that can also be operated without significant external power. An example is the spin-torque microwave detector (STMD) based on magnetic tunnel junctions^17,18,20,30,34–36^, which can operate as an RF detector without the need for external power or external magnetic fields^18,19^. Hence, as a proof of concept demonstration of the potential of self-powered sensor tags, we combined the opto-tag with an MTJ-based STMD to produce an RF-to-optical transducer. Details on the MTJ fabrication are provided in the supplemental information. The STMD consists of two ferromagnetic layers, separated by an ultrathin oxide film. The relative orientation of the magnetization of the two magnetic layers defines the total resistance of the device, referred to as the tunneling magnetoresistance effect (TMR). The resistance is maximum when the two magnetizations are antiparallel and minimum when they are parallel. If an RF signal is applied across the MTJ, the free layer magnetization oscillates at a characteristic frequency in the GHz band in response to spin-transfer torque (STT) and voltage-controlled magnetic anisotropy (VCMA) effects^30,34^. When the excitation frequency is the same as the characteristic frequency, a maximum rectification voltage is generated in the MTJ. While the device can operate in the absence of external bias fields^26^, the characteristic frequency of the device can be altered by applying a local magnetic field, thus tuning the frequency at which maximum rectification voltage is achieved. Due to the similarity of this effect to the rectified voltage generated on microwave diodes, these devices are also known as spin diodes.

**Figure 2:**
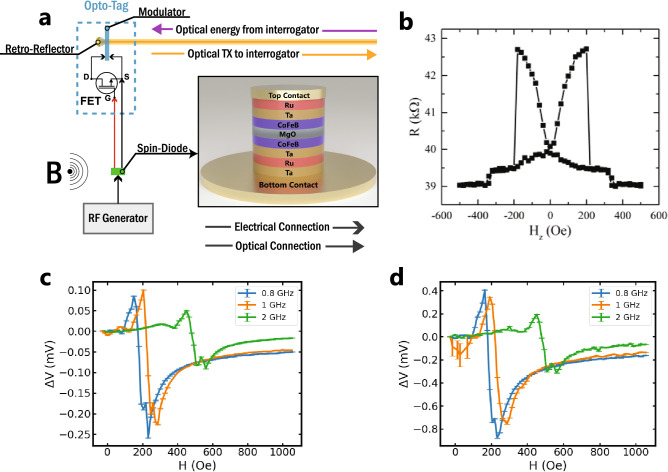
(**a**) System diagram of the spin-diode + opto-tag measurement setup. Inset shows 3D render of spin-diode structure. (**b**) The resistance of the device, as a function of an out-of-plane magnetic field. (**c**) Measured rectified voltage of spin-diode at multiple RF carrier frequencies and bias magnetic field strengths. (**d**) Rectified voltage measured via the opto-tag connected to the output of the spin-diode showcasing a gain of ~ 4.5.

The basic structure of the MTJ used in this work is shown in Fig. 2a. The bottom CoFeB layer was the free layer, featuring an interfacial perpendicular magnetic anisotropy (PMA) originating from the 3d-2p hybridization of Fe and O orbitals at the CoFeB/MgO interface^34,36^. The top CoFeB layer was the in-plane reference layer of the MTJ. Full details of the MTJ based spin diode structure are contained in the supplemental information. The resistance of the device as a function of an out-of-plane magnetic field was measured using a 20 mV sensing voltage and is plotted in Fig. 2b. The plot indicates a full TMR of ~ 10% for this device along with an orthogonal (in-plane reference layer, out-of-plane free layer) configuration at zero field, as expected. In our experiments an external magnetic field (canted 20° with respect to the z axis) was applied by a projected field electromagnet, to tune the resonance frequency of the STMD; however, operation in the same frequency range without this magnetic bias field has previously been demonstrated in STMDs with optimized interfacial PMA^18^.

The rectified voltage was measured with a lock-in amplifier by modulating the amplitude of the input RF carrier signal at 20 Hz. The rectified voltage of the STMD with different applied RF frequencies is shown in Fig. 2c. The RF signals were applied to the STMD device by a RF generator from Keysight ltd model N5171B using a coplanar waveguide Ground-Signal-Ground (GSG) probe from GGB Industries. The output power of the RF generator was fixed at − 6 dBm. The RF frequency was fixed at set values between 0.8 GHz and 2 GHz while the magnetic field strength was swept to shift the resonant frequency of the STMD. To separate the RF power from the DC rectified voltage a bias-tee was used. Using the same probing system, the opto-tag and STMD devices were connected by attaching the opto-tag input to the AC leg of the bias-tee. The rectified voltage measured via the opto-tag interlink is shown in Fig. 2d. An apparent end-to-end gain of ~ 4.5 can be seen between the two signals. The end-to-end gain is ultimately dictated by the gain of the opto-tag device and the interrogator used. In these experiments, the interrogator was an InGaAs free space PIN and an SRS570 transimpedance amplifier. For the system used in this work, the end-to-end gain can be calculated as:

10 $$G_{EE} = \frac{{P_{c} {\Re }G_{in} DOM_{v} }}{S},$$

where $S$ is the amplifier sensitivity in units of A/V. For the system presented here $P_{c}$ = 10E−6 Watts, ${\Re }$ = 0.85 A/W, $G_{in}$ = 18, $DOM_{v}$ = 0.03, $S$ = 2E−6 A/V. These values yield a $G_{EE}$ value of 4.9 which is comparable to the measured gain of ~ 4.5. For these experiments, the distance from the device was fixed at 50 mm.

The output noise of the complete tag and interrogator system was measured with a spectrum analyzer. A 430 µV RMS noise measured at the output was converted to a 97 µV RMS input referred noise (IRF) via the known end-to-end gain. This IRF is higher than the predicted shot noise limited IRF of 65 µV due to some excess white noise that was being produced by our optical source which was a bandpass-filtered superluminescent diode. In general, the choice of the optical source is important to avoid diminished SNR. If the linewidth is too narrow then cavity effects can begin to add considerable noise, if the source linewidth is too broad then it is not efficiently modulated by the SQWM. We note that we have solved these excess noise problems by using a femtosecond pulsed laser source with a 2 nm linewidth.

Since the proposed device can detect a signal carried in the modulation of the amplitude of an RF carrier and transcode it into the modulation of optical intensity, this system is ideal for wireless transmission of information. Information can be encoded on an RF carrier, which simultaneously powers the spin diode (STMD). The induced voltage on the spin diode modulates the opto-tag transmission, which is powered by the same optical beam that is used to interface with it. To illustrate this principle of operation, we used this hybrid opto-spintronic system to transmit three pieces of music: (i) Mahna Mahna, a piece originally by Piero Umiliani and popularized by The Muppet Show, (ii) Johann Sebastian Bach’s Concerto for four harpsichords, strings, and continuo, (iii) Bring it on down by the rock band Oasis. The recordings are included in the supplemental information. Each song was encoded on a RF carrier via amplitude modulation (AM). The carrier frequency during the experiments was 1 GHz, and the power was 0 dBm. In order to tune the spin diode resonance to the frequency of the carrier, an external magnetic field of 200 Oe was applied to the device. The signal was then detected by the opto-spintronic device, and the rectified voltage was optically read at a range of 50 mm using the interrogator system shown in Fig. 2a. Each transmitted song was directly digitized with a DAQ card, without external filters or any other hardware used during the recording.

### Generalized tetherless interlink performance metric

**Figure 3:**
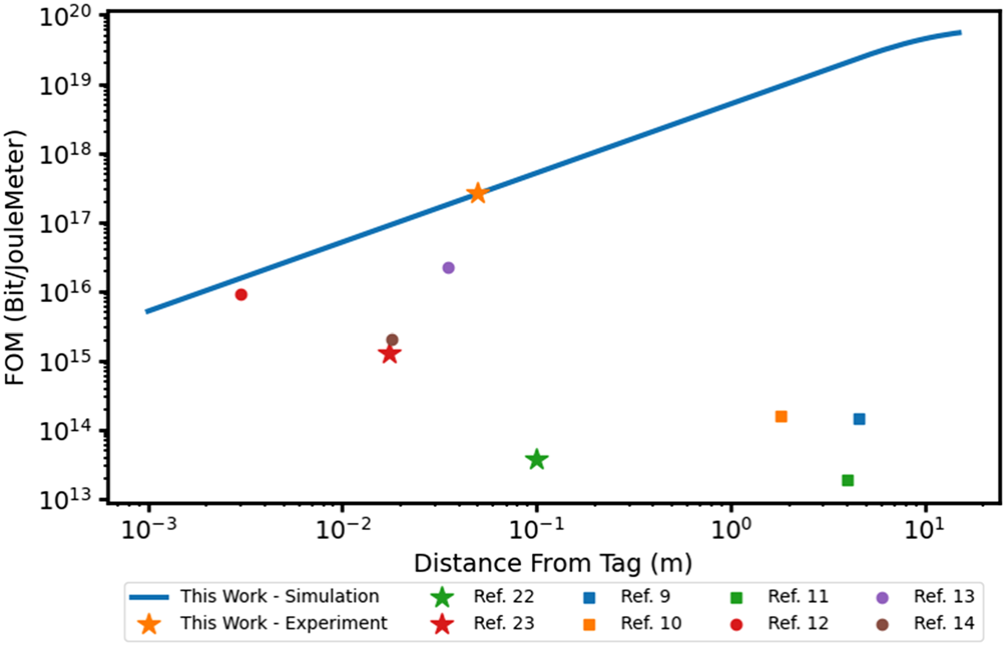
Comparison of RF and optical interlink platforms with the device presented in this work. Circular markers are near-field coupled devices. Squares are far-field RF devices. Stars are optical communication devices.

Comparing the performance of different tetherless interlinks is a difficult task due to the large number of parameters that need to be considered across different technologies; However, all these technologies share a similar goal of producing remote devices that have long communication lengths, low footprint, and high efficiency. Given this, we propose a simple figure of merit that encompasses the core performance metrics of a wireless interlink platform operating at any carrier frequency:

11 $$FoM \propto \frac{L}{{A_{d} E_{bit} }},$$

where $L$ is the communication range, $A_{d}$ is the effective device area, and $E_{bit}$ is the efficiency of the device which can be described by Eq. (4) for an opto-tag. Similarly, for other interlinks $E_{bit}$ will also be a function of the communication range and will scale based on the coupling technique. Using this FoM we can compare the performance of the opto-tag presented here with other electrical and optical interlink technologies. Several recent RF and optically coupled devices compared with the FoM detailed in Eq. (11) are shown in Fig. 3. Each of the compared devices is split into optical (stars), near-field (circles), and far-field (squares) categories. For this FoM, a higher value signifies a device with better overall performance.

The opto-tag performance is calculated by tracking the size of the return beam from a theoretical opto-tag using Eq. (6). The amount of collected power can then be estimated by assuming a gaussian beam and calculating the fraction of the returned power which falls within the collection aperture. The projected performance of the opto-tag assumes an interrogator with a 50 mm collection aperture diameter. The modulated optical signal power ($$P_{s} )$$ is related to the collected power ($$P_{c} )$$ by:

12 $$P_{s} = \left( {DOM_{v} \Delta V_{mod} } \right)P_{c} .$$

The $$DOM_{v}$$ is the fraction of the input optical power that is modulated (depth of modulation) per applied volt on the SQWM. $$\Delta V_{mod}$$ is the voltage swing on the SQWM as defined in Eq. (2). To estimate the full-scale signal to noise ratio ($$SNR_{FS} )$$, $$\Delta V_{mod}$$ is assumed to be equal to the full-scale modulation voltage. As shown by Eq. (1), the full-scale modulation voltage will be dictated by the range of voltages where both the FET and the SQWM maintain a low conductance. The device reported here is capable of producing ~ 150 mV full-scale modulation voltages, this value can be deduced from Supplementary Fig. S2b in the Supplementary Information. The signal current and shot noise current can now be defined as:

13 $$I_{sig} = \frac{{P_{s} {\Re }}}{\sqrt 2 },$$

14 $$I_{shot} = \sqrt {2qP_{c} {\Re }BW} ,$$

where $q$ is the electron charge, ${\Re }$ is the photodetector responsivity, and $BW$ is the system measurement bandwidth. The total system noise current can be defined as:

15 $$I_{noise} = \sqrt {I_{shot}^{2} + I_{amp}^{2} } .$$

$SNR_{FS}$ can then be defined as:

16 $$SNR_{FS} = \frac{{I_{sig} }}{{I_{noise} }}.$$

The effective number of bits in the analog channel is calculated with the $SNR_{FS}$ and Eq. (3). The $FoM$ is then calculated with Eq. (11). As seen in Fig. 3, at longer distances, the FoM begins to fall off. This is due to return power from the opto-tag starting to be lost due to the expansion of the beam as it returns from the opto-tag to the interrogator. As the collected optical signal power is reduced, the amplifier noise will eventually dominate and reduce the SNR below acceptable levels.

**Figure 4:**
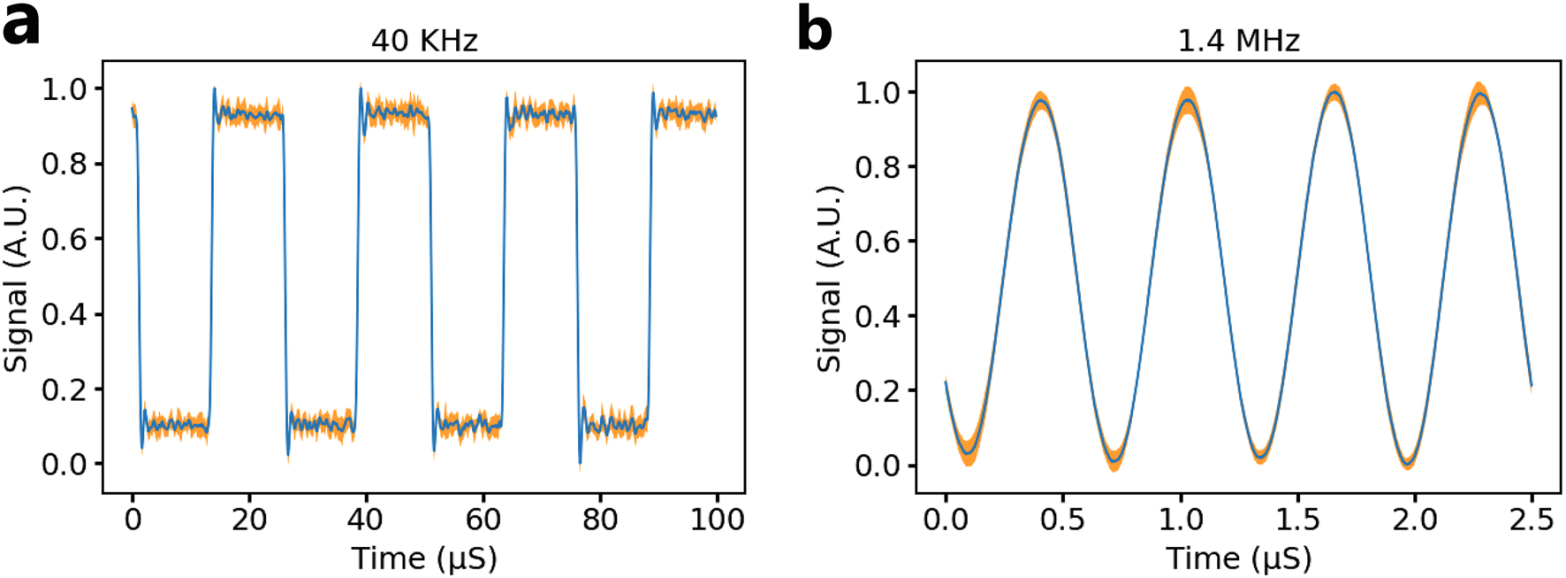
Time domain signals measured at 40 kHz (**a**) and 1.4 MHz (**b**). Both signals were measured at a range of 150 mm with a 20 mm aperture. The blue trace represents the average response to a 45 mV square wave input, calculated with 20 frames. The orange shaded region shows the standard deviation of the signal at that point in each frame, calculated with 20 frames.

The device presented here has a $SNR_{FS}$ of ~ 30 dB up to 1.5 MHz modulation frequency. With knowledge of the SNR at different modulation frequencies, the peak efficiency of the opto-tag can be calculated with Eq. (4) to be ~ 3 pJ/bit or ~ 0.08 fJ/bit/mode at a bit rate of 10 Mb/s. Time domain signals recorded with the opto-tag at 150 mm range with a 25 mm aperture are shown in Fig. 4. at 40 kHz (a) and 1.4 MHz (b) modulation frequencies. In each plot, the blue line is the average signal recorded by the interrogator with a 45 mV square wave at the input of the opto-tag. The orange highlighted zone shows the standard deviation of the signal at that point in the time trace. Both the average response and the standard deviation are calculated with 20 frames.

## Conclusion

A strategy for optically-addressed self-powered devices was presented. By combining a field effect transistor, a stepped quantum well modulator, and a spherical retro-reflector, we demonstrated a millimeter scale free space optical tag which is powered entirely by harvesting power from an input optical beam. Data is encoded on the input optical beam with the SQWM and the optical input is retro-reflected back at the same angle it was incident with. Our fabricated devices display an input gain of ~ 18 and, when combined with an InGaAs PIN and a SRS570 amplifier, the end-to-end gain was measured to be 4.5. The final input referred noise was 97 µV (RMS) with bit rates up to 10 Mb/s at 3 pJ/bit. A figure of merit was presented and used to compare our platform to other available RF solutions. The proposed device was shown to be more performant than any other reported RF-coupled device when considering device size, efficiency, and communication range. The short wavelength of optical signals ensures optimal coupling even as the device size is reduced to micron length scales. We then demonstrated a fully self-powered RF-to-optical transducer by combining the self-powered tag with a spin diode. We demonstrated audio data transfer via this hybrid opto-spintronic link by detecting an injected AM modulated RF signal with the spin diode and transferring the output signal of the spin diode with a free space interlink using the self-powered optical tag at a range of 50 mm.

## Supplementary Information

Supplementary Information 1.

Supplementary Information 2.

Supplementary Information 3.

Supplementary Information 4.
