# Supplementary material for: A 3 pJ/bit free space optical interlink platform for self-powered tetherless sensing and opto-spintronic RF-to-optical transduction
Source: Sci Rep. 2021 Apr 19;11:8504. doi: 10.1038/s41598-021-87885-6 (PMC8055909; doi:10.1038/s41598-021-87885-6)
Supplement: Supplementary file 1 — Supplementary Information 1. [file 41598_2021_87885_MOESM1_ESM.docx]

**A 3 pJ/bit Free Space Optical Interlink Platform for Self-Powered Tetherless Sensing and Opto-Spintronic RF-to-Optical Transduction**

Skyler Wheaton^1^, Victor Lopez Dominguez^1^, Hamid Almasi^1^, Jialin Cai^2^, Zhongming Zeng^2^, Pedram Khalili Amiri^1^, Hooman Mohseni^1^

1. Department of Electrical and Computer Engineering, Northwestern University

2. Suzhou Institute of Nano-Tech and Nano-Bionics, CAS, Suzhou, Jiangsu 215123, People’s Republic of China

**Stepped Quantum Well Modulator (SQWM) Structure and Fabrication:**

The modulator structure was grown via molecular beam epitaxy on a N-doped InP substrate. The QW region is comprised of InGaAsP quantum wells of different compositions, with InAlAs barriers. The QW layers are sandwiched between P and N doped capping layers to produce a PIN like structure for efficient carrier extraction. A 2D cross section of a SQWM is shown in Figure S1a.

The mesa of the modulator is defined using conventional photolithography techniques, combined with a hydrobromic acid etch. The device was passivated with a bi-layer of SiN and SiO_2_ which also acts as 1550 nm anti-reflection (AR) coating. Both bottom and top surfaces were AR coated. Finally, 150 nm gold contacts with a 10 nm titanium adhesion layer and a 20 nm diffusion blocking Mo were fabricated on the top P-doped cap and the N-doped InP substrate.

The stepped quantum wells were designed using a genetic algorithm that has been discussed at length in our previous publication[1].The final result was a structure with 47 quantum wells which produced a total active region thickness of ~1 um. The percent modulation per volt and the transmission of the device is shown in Figure S1b.


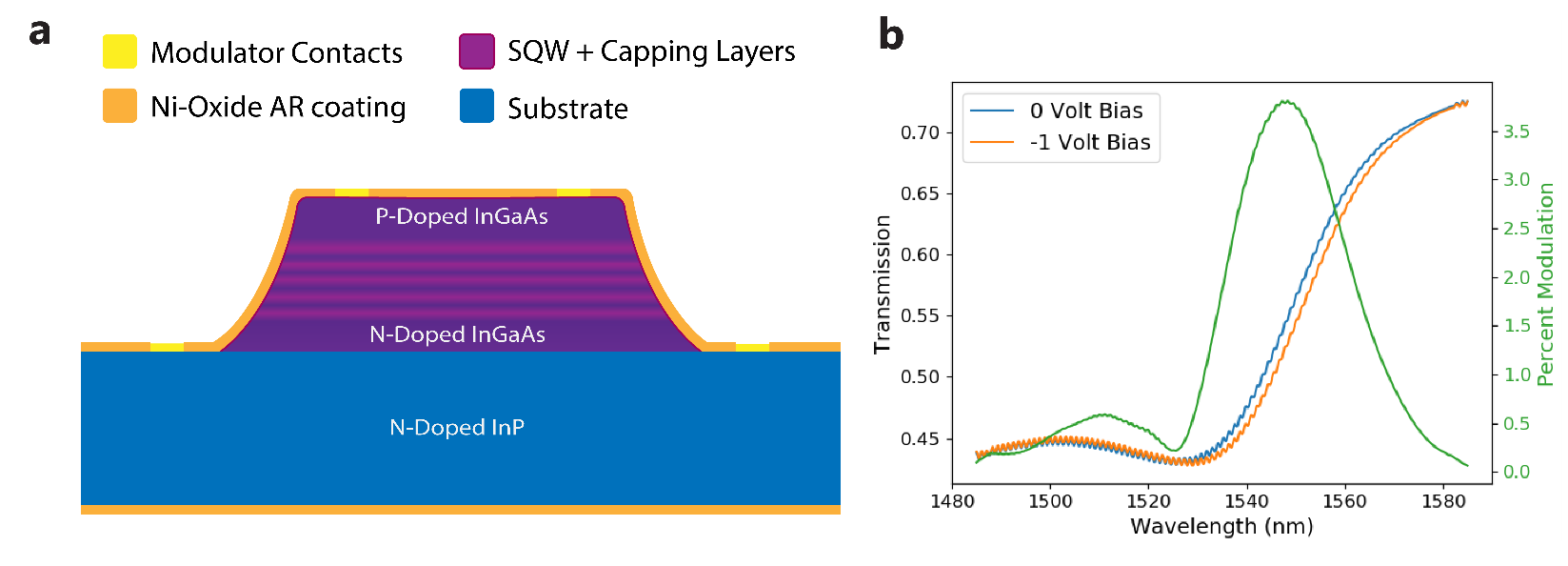


Figure S1. a) 2D cross section of InGaAsP SQWM device. b) Double pass transmission and percent modulation for the 47 QW InGaAsP SQWM used in the demonstrated opto-tag.

**
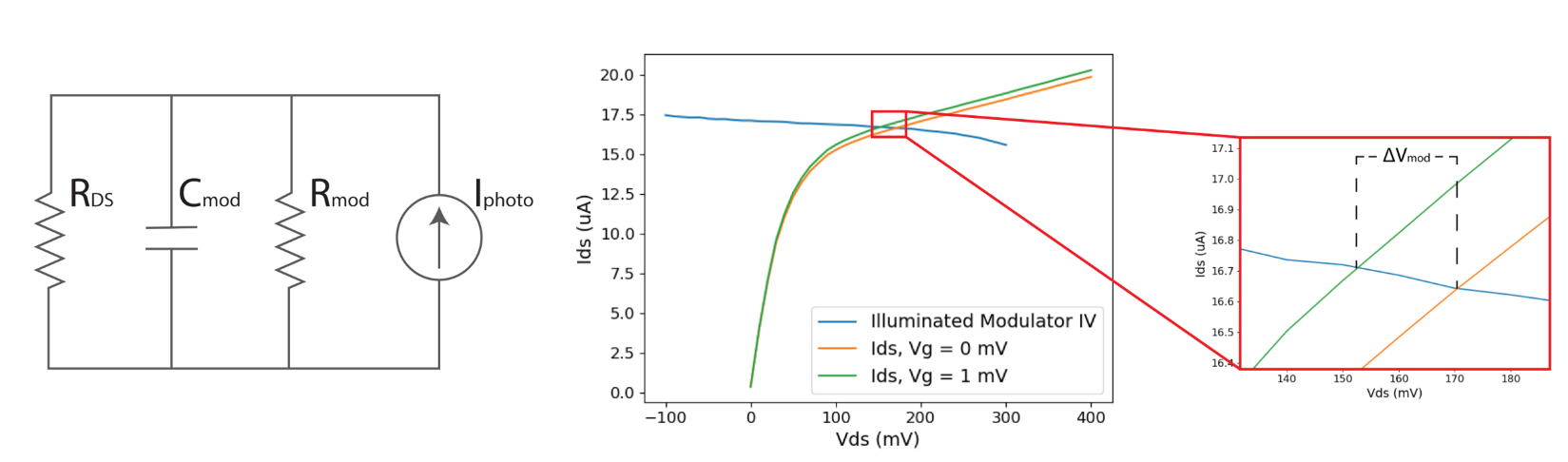
**

Figure 2. a) Simplified circuit model of SQWM+FET. Rds is the resistance of the drain source channel. Cmod is the capacitance of the modulator. Rmod is the differential resistance of the modulator in its linear region. b) Overlay of FET output characteristics and SQWM modulator IV when under illumination with 40 uW of 1550 nm optical power.

**Input gain with FET:**

As discussed in the main text, the gain achieved by combining the FET and the SQWM is ultimately dictated by the transconductance of the FET and the output conductance of both devices. To achieve optimum performance the FET must be in saturation and the voltage across the SQWM must be below the forward bias turn on voltage (~.3 V). When in the linear region for the SQWM, the device can be considered as a capacitor for small signal analysis. Under these conditions the device can be described with the simple circuit shown in Figure S3.

Figure S2a ignores any type of leakage or capacitive coupling to the gate electrode as these effects are minuscule when compared to the generated photocurrent and low V_ds_ values. For this circuit it is clear that the voltage dynamics will be dictated by whichever device has the lowest differential resistance. The input gain produced with this circuit can be directly demonstrated by measuring the output characteristics of the FET at two different gate voltages and stacking this with the IV of an illuminated SQWM to find the operating points of the circuit, as shown in Figure S2b. The inset zooms in on the crossing region of the two curves, showing a change in the modulator bias voltage of almost 20 mV is achieved with a 1 mV change of voltage at the gate.


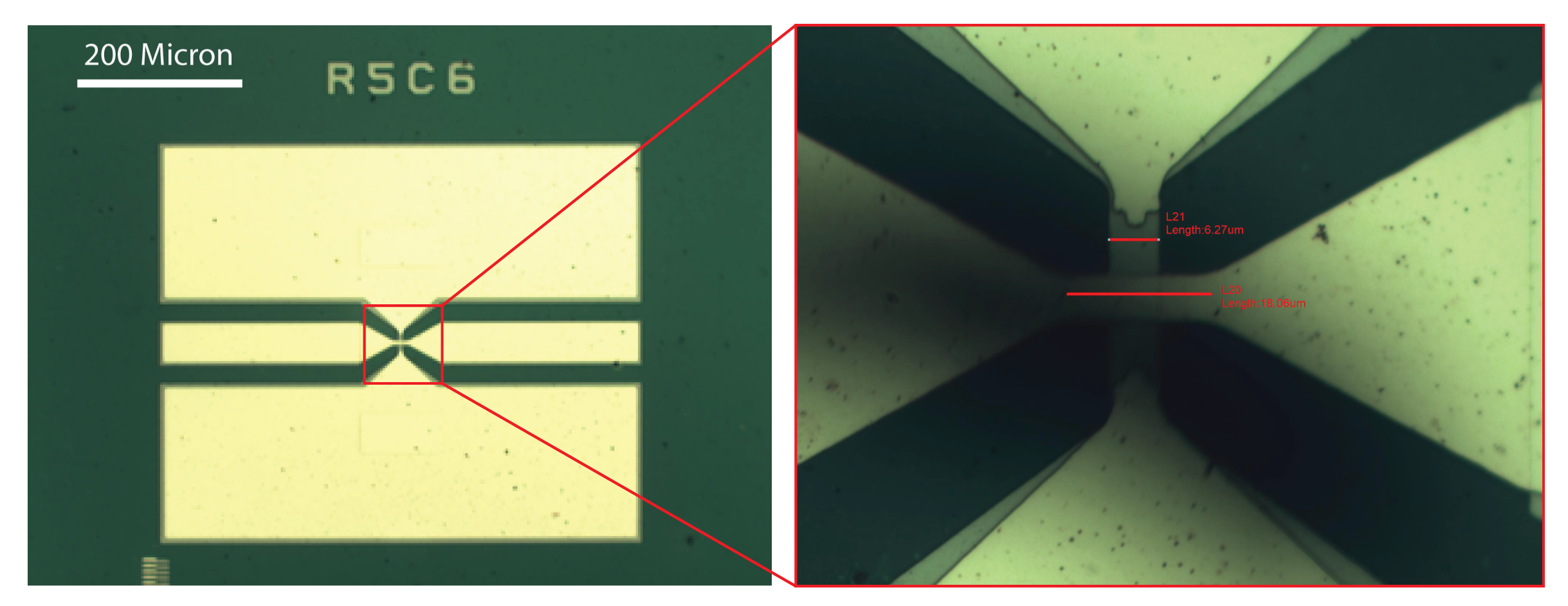


Figure S3. Microscope image of MTJ spin diode with CPW contacts.

**MTJ Spin Diode Structure:**

The MTJ, starting from the substrate, consisted of the following layers: Ta(6) / Ru(10) / Ta(10) / CoFeB(0.85) / MgO(~ 2) / CoFeB(1.5) / Ta(10) / Ru(20) (thickness expressed in nanometers). The bottom CoFeB layer with 0.85 nm thickness was the free layer. The top CoFeB layer (with thickness of 1.5 nm) was the in-plane reference layer of the MTJ. The resulting layer structure was etched into 300 nm × 600 nm elliptical pillars using electron beam lithography. Electrical connections to the top and bottom electrode contacts were made in the form of a Coplanar Waveguide (CPW), to directly apply RF voltage to the MTJ using a ground-signal-ground (GSG) configuration. Microscope image of a completed device is shown in Fig. S5.

**RF to Optical Transduction:**

We encoded audio data via amplitude modulation on a 1 GHz sinusoidal RF carrier. The AM RF signal was applied to the spin diode via direct probing. Power into the spin diode was fixed at 0 dBm. Using the system detailed in Figure 3 a) the rectified voltage from the MTJ was encoded on an optical signal using the opto-tag and interrogated at a range of 50 mm. The optical power incident on the opto-tag was fixed at 75 microwatts. Using this system we transmitted three songs which have been uploaded with this document:

- Transmission i – “Mahna Mahna” a piece originally by Piero Umiliani and popularized by The Muppet Show.
- Transmission ii - Johann Sebastian Bach’s Concerto for four harpsichords, strings, and continuo.
- Transmission iii – “Bring It On Down” by the rock band Oasis.

1. Bianconi, S., et al., *Machine Learning Optimization of Surface-Normal Optical Modulators for SWIR Time-of-Flight 3-D Camera.* IEEE Journal of Selected Topics in Quantum Electronics, 2018. **24**(6): p. 1-8.
